# Strain and Strain Rate Imaging by Echocardiography – Basic Concepts and Clinical Applicability

**DOI:** 10.2174/157340309788166642

**Published:** 2009-05

**Authors:** Michael Dandel, Hans Lehmkuhl, Christoph Knosalla, Nino Suramelashvili, Roland Hetzer

**Affiliations:** Department of Cardiothoracic and Vascular Surgery, Deutsches Herzzentrum Berlin, Germany

**Keywords:** Strain imaging, echocardiography, myocardial contraction, diagnosis, prognosis.

## Abstract

Echocardiographic strain and strain-rate imaging (deformation imaging) is a new non-invasive method for assessment of myocardial function. Due to its ability to differentiate between active and passive movement of myocardial segments, to quantify intraventricular dyssynchrony and to evaluate components of myocardial function, such as longitudinal myocardial shortening, that are not visually assessable, it allows comprehensive assessment of myocardial function and the spectrum of potential clinical applications is very wide. The high sensitivity of both tissue Doppler imaging (TDI) derived and two dimensional (2D) speckle tracking derived myocardial deformation (strain and strain rate) data for the early detection of myocardial dysfunction recommend these new non-invasive diagnostic methods for extensive clinical use. In addition to early detection and quantification of myocardial dysfunction of different etiologies, assessment of myocardial viability, detection of acute allograft rejection and early detection of allograft vasculopathy after heart transplantation, strain and strain rate data are helpful for therapeutic decisions and also useful for follow-up evaluations of therapeutic results in cardiology and cardiac surgery. Strain and strain rate data also provide valuable prognostic information, especially prediction of future reverse remodelling after left ventricular restoration surgery or after cardiac resynchronization therapy and prediction of short and median-term outcome without transplantation or ventricular assist device implantation of patients referred for heart transplantation.

The Review explains the fundamental concepts of deformation imaging, describes in a comparative manner the two major deformation imaging methods (TDI-derived and speckle tracking 2D-strain derived) and discusses the clinical applicability of these new echocardiographic tools, which recently have become a subject of great interest for clinicians.

## INTRODUCTION

Although conventional echocardiography is considered to be reliable for ventricular wall motion analysis and assessment of regional myocardial function, the visual estimation of wall motion is very subjective and therefore highly operator dependent. It also has high interobserver and intraobserver variability and allows only limited evaluation of radial displacement and deformation, without the possibility of assessing myocardial shortening and twisting [1,2].

During recent years, velocity imaging, displacement imaging and deformation imaging (strain and strain-rate imaging) have emerged as valuable tools for more comprehensive and reliable echocardiographic assessment of myocardial function [2-11]. 

## BASIC CONCEPTS AND TERMINOLOGY

For a better understanding of different echocardiographic modalities available for the assessment of myocardial contractile function, it is important to make a distinction between myocardial wall motion and wall deformation [3-7]. Whereas velocity and displacement characterize wall motion, strain and strain-rate describe wall deformation. Over time a moving object will change its position (displacement) but does not undergo deformation if all its parts move with the same velocity. If, however, different parts of the object move with different velocities, the object will undergo deformation and will change its shape. Thus wall motion measurements (displacement and velocity) cannot differentiate between active and passive movement of a myocardial segment, whereas deformation analyses (strain and strain-rate imaging) allow discrimination between active and passive myocardial tissue movement. 

The term “strain”, which in everyday language can mean “stretching”, is used in echocardiography to describe “deformation” [12].

However the concept of strain is complex. Thus for a one-dimensional (1D) object (i.e. an infinitesimally thin bar) the only possible deformation is lengthening or shortening and the linear strain (amount of deformation) can be defined by the formula: 

$\varepsilon =\frac{\text{L}-{\text{L}}_{0}}{{\text{L}}_{0}}=\frac{\Delta \text{L}}{{\text{L}}_{0}},$

where **ε** = strain, L_0_ = baseline length and L = instantaneous lengths at the time of measurement. 

When the length of the object is known not only before and after deformation, but also during the deformation process the instantaneous strain can be defined as: 

$\varepsilon \left(\text{t}\right)=\frac{\text{L}\left(\text{t}\right)-\text{L}\left({\text{t}}_{0}\right)}{\text{L}\left({\text{t}}_{0}\right)},$

where L(t) is the length at the time instance t and L(t_0_) ≡ L_0_. The instantaneous deformation is thus expressed relative to the initial length (Lagrangian strain) [12]. The deformation can also be expressed relative to the length at a previous time instance (natural strain) and in this definition of instantaneous strain the reference value is not constant over the time but changes during the deformation process [12]. For small deformations the Lagrangian and natural strain are approximately equal whereas for large deformations which can occur during ventricular contraction and relaxation the difference between Lagrangian and natural strain are relevant. For myocardial strain measurements it appears more appropriate to measure the natural strain because the measured values are less dependent on the definition of the initial length L_0_ [12].

For two-dimensional (2D) objects, the deformation is not limited to lengthening or shortening in one direction. A 2D object can lengthen or shorten along the x or y axis (normal strain) and can also distort (shear strain) by the relative displacement of the upper to the lower border or the right border to the left border [12]. Thus, in two dimensions strain has four components, two normal strains and two shear strains. More complex is the deformation of three-dimensional (3D) objects such as myocardial segments. In this case there are three normal strains (along the x, y and z axes) and six shear strains. To completely define the deformation of 3D objects, all nine strain components must be defined. Today, echocardiographic deformation imaging allows 1D measurements based on tissue Doppler imaging and 2D strain measurements based on speckle-tracking imaging. 

The amount of deformation (positive or negative strain) is usually expressed in %. Positive strain values describe thickening, negative values describe shortening, of a given myocardial segment related to its original length. During myocardial contraction, as the wall shortens it also thickens and thus assessment of all parameters, radial thickening (positive strain), circumferential shortening (negative strain) and longitudinal shortening (negative strain), is useful for the evaluation of contractile function. 

Strain rate (SR) is the rate by which the deformation occurs (deformation or strain per time unit). The unit of strain rate is s^-1^ and the local rate of deformation or strain per time unit equals velocity difference per unit length:

$\varepsilon =\frac{\Delta \varepsilon }{\Delta \text{t}}=\frac{\left(\Delta \text{L}/{\text{L}}_{0}\right)}{\Delta \text{t}}=\frac{\left(\Delta \text{L}/\left(\Delta \text{t}\right.\right)}{{\text{L}}_{0}}=\frac{\Delta \text{V}}{{\text{L}}_{0}},$

where ΔV is the velocity gradient in the segment studied. Thus, the velocity gradient (i.e. difference in velocities between two points of the myocardial wall) can be used for SR calculations. The SR has the same direction as the strain (negative strain during shortening and positive strain during elongation). 

Ventricular wall motion (velocity and displacement) is position dependent. Thus, as the apical parts of the ventricle pull down the ventricular base, the wall motion velocity and wall displacement increase from apex to base and some of the motion in the base is an effect of apical contraction – tethering. Thus, even completely passive segments, without deformation, can show motion [5]. 

Myocardial deformation (strain and SR) is more constant along the ventricular wall (position independent if the velocity gradient is evenly distributed). Therefore, strain and SR imaging (deformation analysis) is more useful than wall motion analysis (velocity and displacement) for detection of regional myocardial dysfunction [5,7]. Nevertheless, because of the relationship between myocardial motion and deformation, wall motion velocity measurements by tissue Doppler can be used to obtain regional and global strain (and SR) data [3-7,9,10].

It is important to know that, although strain and SR are particularly suited for the assessment of systolic function (especially regional contractile function), they are not measurements of contractility because deformation is load dependent. Contractility (the basic property of the myocardium that reflects its active state, rather than loading conditions) is reflected by the stress / strain relation [13]. The relation to contractility of the different parameters used to evaluate systolic function can differ. Thus, because the final part of ejection occurs by inertial effects after myocyte contraction is finished, peak systolic strain rate, being an early systolic event, is more closely related to contractility than the ejection fraction (EF) [5]. 

## GENERAL PRINCIPLES OF ECHOCARDIOGRAPHIC DEFORMATION IMAGING 

Initially myocardial deformation imaging became possible using tissue Doppler [4]. More recently myocardial deformation imaging also become possible with myocardial speckle tracking using 2D echocardiography [15]. Figs. (**1**) and (**2**) show examples of myocardial deformation imaging using tissue Doppler and 2D speckle-tracking, respectively. The examples in these 2 figures also show the advantages of strain imaging in comparison to velocity and displacement imaging in the evaluation of regional myocardial contractile function.

### Tissue Doppler-Derived Strain and Strain-Rate Imaging

Tissue Doppler imaging (TDI), also known as tissue velocity imaging (TVI), is currently accepted as a sensitive and sufficiently accurate echocardiographic tool for quantitative assessment of cardiac function [3,5-11]. Several tissue Doppler velocity parameters appeared to be useful for the diagnosis and prediction of long-term prognosis in major cardiac diseases [8,10,11]. Myocardial time-velocity curves can be obtained either online as spectral pulsed TDI, known as pulsed wave TDI (PW-TDI), or reconstructed offline from two-dimensional (2D) color coded TDI images, known as color TDI (C-TDI) loops. In addition to velocity and displacement (tissue tracking) measurements, due to the relationship between velocity and strain rate, TDI also allows the reconstruction of strain (and strain rate) curves and color coded images. Thus, the transmural velocity gradient (difference in endocardial and epicardial velocities divided by the instantaneous wall thickness) is equal to the transmural strain rate (rate of wall thickening), whereas the longitudinal velocity gradient over a segment with a fixed distance is a measure of longitudinal strain rate. Before the development of color coded TDI, it was difficult to distinguish myocardial contraction from translational motion of the heart. Thus, during systole, in addition to radial wall thickening and longitudinal wall shortening, the left ventricle (LV) also rotates about its long axis and translates anteriorly. Calculation of myocardial velocity gradients (MVG) allows the assessment of wall motion independently from the translational motion of the heart [3,5,14,15]. However, as shown by the equation for tissue Doppler derived MVG:

$\mathrm{MVG}\left({\text{s}}^{-1}\right)=\left({\text{V}}_{2}-{\text{V}}_{1}\right)/\text{d}•\cos\theta$

where V_2_ - V_1_ is the difference in velocities, d is the distance between the two points of velocity measurement and cos θ is the cosine of the angle between the ultrasound beam and the direction of myocardial movement, all tissue Doppler derived data on wall motion and deformation are angle dependent. Thus, for acceptable calculations, an angle deviation below the 20 to 15 degrees is mandatory. It is important to be aware that assessing tissue movement in relation to the transducer rather than relative to adjacent segments is a fundamental limitation of tissue velocity imaging, which can also affect tissue Doppler derived strain (and strain rate) imaging [11].

Although strain and strain rate (SR) measurements derived from the myocardial velocities are promising for the evaluation of ventricular contractile function they also have disadvantages [16]. First of all they are derived from 1-dimension velocity measurements while, as already mentioned, the myocardium deforms simultaneously in three dimensions. An important disadvantage relates to limits on spatial resolution that are imposed by imaging at high temporal resolution. Other disadvantages of the TDI-derived strain and SR imaging technique are the time consuming steps for data acquisition and processing and the necessity of expert readers. It is also important to know that the comparison of adjacent velocities is highly sensitive to signal noise and the signal-to-noise ratio of TDI-derived SR measurements is reduced. This can be improved by increasing sample distance but only in exchange for lower spatial resolution. 

Taking into account all the aspects mentioned above it is not surprising that TDI-derived strain and SR measurements are not highly reproducible (more than 10-15% interobserver variability). This is one of the explanations why this technique has not become standard in daily praxis. However, in the hands of very experienced and highly trained operators this method can be a valuable non-invasive tool for routine clinical use to evaluate the myocardial contractile function. Despite all limitations this technique has been initially validated with sonomicrometry and also with magnetic resonance imaging [7,9].

### Non-Doppler Speckle-Tracking Derived 2D-Strain Imaging

Non-Doppler 2D-strain imaging derived from speckle tracking is a newer echocardiographic technique for obtaining strain and SR measurements [2,17-21]. It analyzes motion by tracking speckles (natural acoustic markers) in the 2D ultrasonic image. These acoustic markers are statistically equally distributed throughout the myocardium and their size is about 20 to 40 pixels. These markers (“stable” speckles) within the ultrasonic image are tracked from frame to frame. Special software allows spatial and temporal image processing with recognition and selection of such elements on ultrasound images. The geometric shift of each speckle represents local tissue movement. When frame rate is known, the change in speckle position allows determination of its velocity. Thus, the motion pattern of myocardial tissue is reflected by the motion pattern of speckles. By tracking these speckles, strain and strain rate can be calculated. The advantage of this method is that it tracks in two dimensions, along the direction of the wall, not along the ultrasound beam, and thus is angle independent [2]. The 2D echocardiographic loops obtained from pararasternal and apical views are processed offline. This requires only one cardiac cycle to be acquired but strain and SR data can be obtained only with high resolution image quality at high frame rate [2,16]. The necessity of high image quality is a major limitation for routine clinical applicability in all patients. At present, the optimal frame rate for speckle-tracking seems to be 50-70 frames per second (FPS), which is lower compared to TDI (>180 FPS). This could, however, result in under-sampling, especially in patients with tachycardia and also during strain and SR measurements performed throughout stress echocardiography. Moreover, rapid events during the cardiac cycle such as isovolumetric phases may not appear on images and peak SR values may be reduced due to under-sampling, in isovolumetric phases and in early diastole. Using higher frame rates could reduce the under-sampling problem, but this will result in a reduction of spatial resolution and consequently less than optimal region of interest (ROI) tracking [22]. Low frame rate increases the spatial resolution, but because speckle-tracking software uses a frame-by-frame approach to follow the myocardial movement and searches each consecutive frame for a speckle pattern closely resembling and in close proximity to the reference frame, with too low a frame rate the speckle pattern could be outside the search area, again resulting in poor tracking [23,24]. It is also important to know that different tracking algorithms potentially produce different results and therefore it should be kept in mind that a periodical update of the software package conceivably influences reference values.

Although speckle-tracking derived 2D-strain and TDI-derived strain calculations do not give the same values (2D-strain imaging gives lower SR values), strain and SR measurements obtained by these two different imaging techniques correlate well [2]. For the LV, the reproducibility of 2D-strain measurements is better than that of TDI-derived strain measurements. The intraobserver and interobserver variability for 2D-strain and SR measurements were found to be low: 3.6% to 5.3% and 7% to 11.8%, respectively [2]. Ingul *et al. *found lower interobserver variability for non-Doppler 2D-strain measurements in comparison to TDI-derived strain measurements and automated non-Doppler 2D-strain measurements also appeared significantly less time consuming [18]. The lack of angle dependency is a great advantage of non-Doppler 2D-strain imaging in comparison to TDI-derived strain data. 

## CLINICAL USEFULNESS AND FUTURE DIRECTIONS OF ECHOCARDIOGRAPHIC DEFORMATION IMAGING 

Measurements of strain and strain rate by echocardiography have been validated using microcrystals and magnetic resonance imaging [16,25-27]. Comparing non-Doppler 2D-strain imaging with tagged magnetic resonance imaging (the current “gold standard” for deformation analysis) non Doppler 2D-strain measurements correlated well with data obtained by magnetic resonance imaging, both in normal myocardial segments and infarcted areas (r = 0.87, P<0.001) [25]. Experimental work performed on adult dogs showed that global diastolic strain rate can be useful for the assessment of ventricular relaxation and estimation of filling pressures [28]. In a study on 137 consecutive patients with suspected congestive heart failure of different etiologies it was also shown that mean longitudinal LV strain is closely related to plasma brain-type natriuretic peptide (BNP) levels, in patients with both systolic and diastolic heart failure [29]. 

Strain and strain rate measurements appeared to be sensitive indicators for sub-clinical diseases, including diabetes, systemic sclerosis, myocardial ischemia, arterial hypertension, isolated mitral regurgitation, aortic regurgitation and non-ischemic cardiomyopathies, and also very useful for the assessment of myocardial damage after infarction, evaluation of myocardial revascularization efficiency and prediction of patient outcome with heart failure [11,30-46]. Early detection of myocardial involvement in asymptomatic patients with systemic sclerosis, diabetes, amyloidosis, Duchenne’s progressive muscular dystrophy and Kawasaki syndrome is an important indication for strain and strain-rate imaging [47-51]. Recently it was also found that 2D-strain imaging is highly sensitive for the early detection of doxorubicin induced cardiac injury, and radial strain reduction in patients who underwent chemotherapy with doxorubicin appeared to be associated with histologic markers of doxorubicin cardiomyopathy [52]. 

Strain and strain rate assessment also appear to be useful in sports medicine for the quantification of LV systolic function in athletes involved in sports requiring endurance or strength and the differentiation of physiologic hypertrophy in athletes’ hearts from asymptomatic nonobstructive hypertrophic cardiomyopathy, which is the major cause of sudden cardiac death in young competitive athletes [53-55]. Strain imaging may also be used to differentiate physiologic cardiac hypertrophy (“athlete’s heart”) from hypertensive cardiac hypertrophy [56]. 

Recently it was shown that strain and strain-rate imaging is also useful for the evaluation of right ventricular (RV) function in pulmonary hypertension and RV diseases of different etiologies (RV infarction, arrhythmogenic RV dysplasia/cardiomyopathy) [57-63]. 

The clinical usefulness of echocardiographic strain and SR imaging in children is another important aspect, especially because in these patients the impact of heart rate (HR) on strain and SR measurements is more evident than in adults. It has been shown that HR changes in healthy children during growth have an important impact on both systolic and diastolic myocardial strain and also on late diastolic SR calculated from color Doppler myocardial imaging [64]. The impact of high heart rates as already mentioned on 2D-strain and SR measurements is of special importance in pediatric patients. Therefore, for the evaluation of regional and global myocardial deformation in children, HR at rest should be considered an important factor.

The assessment of myocardial viability is one of the most important clinical indications for echocardiographic strain and strain-rate imaging. TDI measurements in dogs with experimental occlusion of the left anterior descending (LAD) or circumflex (Cx) coronary artery showed that diastolic strain rate during dobutamine infusion reliably identified segments with >20% transmural infarction and, in comparison to other TDI-derived parameters, it related best to the extent of interstitial fibrosis (r = -0.86; P<0.01) [65]. The validity of non-Doppler 2D-strain imaging for identification and quantification of myocardial ischemia was also proved experimentally in pigs with occlusion of the LAD and in rat ischemia-reperfusion models with temporary LAD occlusion [66,67]. Also it was experimentally shown that speckle-tracking 2D-strain imaging correctly identifies segmental LV dysfunction induced by the scarring that follows myocardial infarction in rats [68]. Both non-Doppler 2D-strain imaging and TDI-derived strain imaging were also used successfully in clinical diagnosis for detection of LV myocardial ischemia and infarction and estimation of myocardial infarction size [32-39]. Strain and strain rate measurements obtained by non-Doppler 2D-strain imaging were found to be highly sensitive and specific for the diagnosis of myocardial infarction [2]. In a study on 30 patients Leitmann *et al. *found that 80.3% of the infarcted segments and 97.8% of normal segments were adequately recognized by speckle tracking based 2D-strain imaging [17]. Comparing the strain and strain rate obtained by speckle tracking based 2D-strain imaging with those obtained in the same patients by TDI, the authors found no significant differences. Comparing the accuracy of 2D-strain imaging derived from speckle tracking with TDI-derived strain imaging in 150 patients undergoing dobutamine stress echocardiography (DSE) and coronary angiography, Hanekom *et al. *found similar accuracy of these two methods during DSE in the anterior coronary circulation [69]. However, in the same study the accuracy of 2D-strain rate measurements for the diagnosis of right coronary artery (RCA) stenosis, was lower than that of TDI-derived strain rate measurements. Strain and strain rate measurements also appeared useful for detection of regional myocardial dysfunction in patients with right ventricular (RV) myocardial infarction [69].

Augmentation of strain and strain rate with dobutamine is a marker of myocardial viability and it was shown that deformation parameters obtained by both non-Doppler speckle tracking (2D-strain imaging) and TDI can improve the diagnostic and prognostic assessment of myocardial ischemia and post-infarction scars during dobutamine stress echocardiography [11,31,70-73]. In patients with chronic ischemic LV dysfunction it was shown that combined assessment of long-axis and short-axis function using 2D-strain imaging may be used to identify the transmural extent of myocardial infarction [70].

**Fig. (3):**
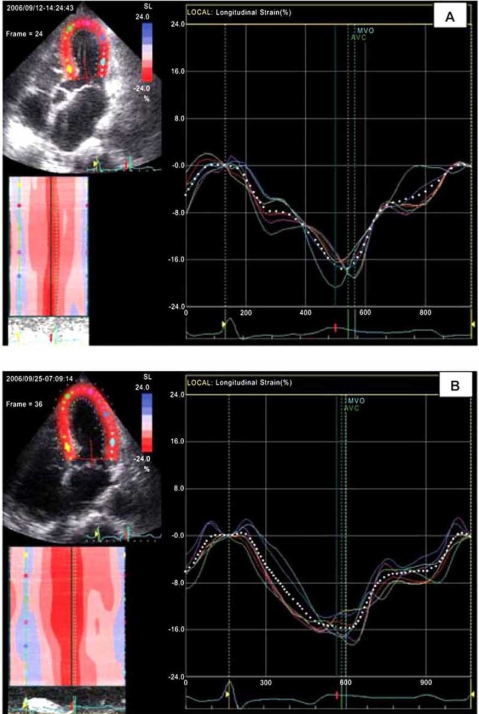
Longitudinal global strain (dotted white curve) and regional longitudinal strain curves (distinctively colored curves for 6 left ventricular wall  segments) obtained from apical 4-chamber views by speckle-tracking 2D-strain imaging in a patient with Takotsubo cardiomyopathy. Although during catecholamine induced severe LV dysfunction with apical ballooning **(A)** the apex appeared nearly akinetic (no visible relevant inward movement) the longitudinal strain curves showed the same uniform longitudinal shortening as after recovery **(B)**, when also visually no regional wall motions were detectable. Thus, the visual analysis of inward movement used in conventional echocardiographic examinations can be misleading in the evaluation of regional myocardial contraction because it can not exclude the existence of longitudinal shortening (not visible with the naked eye) in the apparently akinetic region [cf. Dandel *et al.* International Journal of Cardiology 2008].

Important discrepancies were found in patients with coronary stenoses when comparing the results of visual wall motion assessment with those obtained by deformation analysis [2]. The main reason for these discrepancies is well known: the fact that visual assessment of wall motion in four chamber views relies mainly on evaluation of inward motion of the myocardium (the transverse component of contraction), whereas deformation imaging allows the evaluation of functional components such as longitudinal myocardial shortening, which are barely visible to the naked eye [2]. Recently we published our observations on 2 patients with stress cardiomyopathy (Takotsubo cardiomyopathy) in which for the first time cardiac function was evaluated also by 2D-strain imaging [74]. During stress/catecholamine induced LV dysfunction, wall motion analysis performed by 2D-strain imaging revealed in both patients uniform systolic myocardial longitudinal shortening despite the typical ballooning and akinetic appearance of the LV apex and the hyperkinetic movement of the LV base, indicating myocardial viability in the visually nearly akinetic apical region and also questioning the existence of relevant differences in regional myocardial contractility. During maximal LV dysfunction, due to the particular geometry (larger diameters and thinner walls in apical regions), the systolic circumferential wall stress (**σ**_c_) was several times higher in the apical regions (apical/basal systolic **σ**_c_-ratios up to 7.3), high enough to oppose circumferential fiber shortening and consequently also high enough to prevent the visible inward wall motion in the apical region. At the same time, as revealed by 2D strain imaging, longitudinal shortening, which is usually not visible with the naked eye, was not affected (Fig. **3**). Our findings might be important not only for the explanation of wall motion in stress cardiomyopathy but because they also reveal the potential impact of myocardial deformation analysis on the pathophysiological understanding of myocardial function in relation to ventricular geometry regardless of the nature of cardiac diseases. 

The advantages provided by deformation analysis can improve the decision making in patients referred for cardiac surgery and, in our experience, 2D-strain imaging is indeed a valuable tool for evaluation of patients before and after cardiac surgery. Fig. (**4**) shows an example of longitudinal strain images obtained in a patient before and after coronary bypass operation combined with mitral valve reconstruction. 

In our department, 2D-strain imaging is also the method of choice for patient selection for surgical ventricular restoration (SVR) to improve the LV function after severe myocardial infarction. We also found that systolic dyssynchrony and the end-systolic dyssynergy indexes, calculated from regional strain values, are highly sensitive for evaluations of myocardial functional changes during the postoperative reverse remodeling processes after SVR [40,75]. Figs. (**5**) and (**6**) show examples of 2D-strain and strain-rate recordings obtained before and after SVR. 

Tissue Doppler derived strain and strain rate measurements are also useful for the monitoring of LV function during the reverse remodeling processes after aortic valve replacement in patients with aortic stenosis [76].

Before myocardial deformation imaging became available it had already been shown that in heart transplant recipients TDI wall motion assessment is useful for rejection diagnosis and early detection of patients with relevant transplant coronary artery disease (TxCAD) [77-79]. In our department TDI has been routinely used since 1998 and became a cornerstone for the monitoring of cardiac allograft function and for the timing of follow-up myocardial biopsies and coronary angiographies. After 2D-strain imaging became available, its usefulness for post-transplant follow-up monitoring of cardiac function was also investigated [40,80-82]. Comparing the deformation parameters obtained from patients who underwent routine endomyocardial biopsies, Marciniak *et al. *found significantly lower LV longitudinal and radial peak systolic strain and strain rate values in patients with acute rejection ≥ grade 1B in comparison to those with biopsies graded between 0 and 1A [82]. In patients with biopsy-proven acute rejection episodes ≥ grade 3, we found a significant (p<0.05) reduction of LV systolic and  diastolic radial,  circumferential  and longitudinal global peak strain and strain rate values in comparison to the values measured before rejection. The same changes were also detected in patients with cellular rejections grade ≤ 2 who had clinical symptoms and/or additional immune-histological signs of vascular (humoral) rejection. An example of myocardial strain changes during acute rejection is shown in Fig. (**7**). Systolic and diastolic global strain rate reduction appeared to be more sensitive for the early detection of acute rejection than the reduction of systolic and early diastolic global strain values. As shown in Fig. (**8**), even mild acute rejection (grade 1) in completely asymptomatic patients, without any change in conventional echocardiographic parameters, can be associated with relevant strain rate changes. A sudden drop of ≥15% of the radial global strain rate in heart transplanted patients appeared highly predictive for acute biopsy proven rejection [80]. Two-dimensional strain imaging is also useful for the evaluation of anti-rejection treatment efficacy. In patients without visible alterations in LV kinetics, 2D-strain  imaging also appeared reliable for non-invasive prediction of TxCAD with  and without focal stenoses (>50% narrowing) of main epicardial coronary arteries [40,83,84]. Eroglu *et al. *found that strain and strain-rate imaging in combination with dobutamine stress echocardiography is useful for early detection of TxCAD before the development of relevant stenoses detectable with conventional angiography [84]. The high predictive value for coronary stenoses of systolic strain dyssynchrony and dyssynergy indexes found in our patients even at rest (positive and negative predictive values of 90%-95% and 91-97%, respectively) recommended 2D-strain imaging as a non-invasive tool with the potential to facilitate early detection of stenoses and to enable angiographies to be timed, sparing patients frequent routine heart catheterizations [40]. Fig. (**9**) shows examples of strain and strain rate images obtained from heart transplant recipients with focal stenoses of the coronary arteries.

The negative effects of altered electrical activation on ventricular mechanical function were already recognized more than 40 years ago [85]. More recently this aspect has gained important scientific interest and several large clinical trials have established the long-term benefits of cardiac resynchronization therapy (CRT) in patients who have severe LV dysfunction and a wide QRS complex [86,87]. However, despite these promising results, approximately 30% of patients selected on the basis of QRS duration do not respond to CRT and there is increasing evidence that the main predictor of responsiveness to CRT is mechanical rather than electrical dyssynchrony [87,88]. Measurement of regional myocardial electro-mechanical events with velocity data acquired with tissue Doppler imaging facilitate identification of mechanical dyssynchrony and has been shown to be useful to select patients who may better respond to CRT [88]. However, identification of responders by time-delay indexes alone is limited, especially in patients with ischemic cardiomyopathy who have myocardial segments with delayed contraction, which is often caused by scar [88]. Two-dimensional strain imaging by speckle tracking and TDI-derived strain imaging are well suited to detecting and defining intraventricular dyssynchrony and they have already proved to be useful for both the selection of patients who might benefit from cardiac resynchronization therapy (CRT) and the evaluation of CRT efficiency [83,84,89-94]. The accuracy of speckle tracking 2D-strain echocardiography in the detection and quantification of cardiac dyssynchrony was validated experimentally in canine and sheep models [95,96]. In a canine model of dyssynchrony with and without heart failure, Arita *et al. *found radial strain by speckle tracking to be more accurate than TDI velocity to detect cardiac dyssynchrony [97]. Nevertheless, whereas parameters of systolic dyssynchrony based on TDI longitudinal and radial velocity measurements were able to predict the efficacy of CRT in patients with heart failure, parameters of systolic dyssynchrony based on longitudinal and radial strain data obtained from TDI and speckle tracking 2D-strain imaging appeared not predictive for CRT results [91,94]. However, the combination of parameters of systolic dyssynchrony based on TDI longitudinal velocity with parameters of systolic dyssynchrony based on radial strain data obtained by speckle-tracking (2D-strain imaging) showed the highest predictive value for LV functional response to CRT, which was significantly better than that of either technique alone (p<0.0001) [98]. Recently, the value of strain imaging for prediction of future reverse remodelling after CRT has been improved by the development of a strain delay index calculated by use of longitudinal strain assessed by 2D speckle tracking [88]. At a cut-off value of ≥25% the strain delay index showed high positive and negative predictive values (90% both) for response to CRT [88]. Also recently it was shown that the incorporation of local 2-D echocardiographic deformation data into a 3-D model by dedicated software allows a comprehensive analysis of spatio-temporal distribution patterns of myocardial dyssynchrony of the global LV deformation and the development of new indexes that may better reflect myocardial dyscoordination and/or impaired ventricular contractile efficiency [99]. An important aspect for CRT effectiveness is its dependency on the LV lead position. To find out the optimal LV lead position is therefore a major goal, and recent studies have shown that 2D-strain imaging is a useful tool for this purpose [91]. 

Mechanical dyssynchrony of the LV is a more sensitive marker of myocardial dysfunction than the ejection fraction (LVEF) [93,96]. In patients with idiopathic dilated cardiomyopathy who were accepted for heart transplantation (HTx) we found that systolic and diastolic LV dyssynchrony and dyssynergy, which were detectable by 2D-strain imaging in all investigated patients, were more closely related to hemodynamic alterations, exercise intolerance and patient outcome than LVEF [100]. We also found that 2D-strain imaging provides prognostic information, which can be useful for patients’ selection for HTx. Thus, in patients with similar LVEF, those with rapid worsening toward inotropic support dependence showed higher dyssynchrony and lower global strain rate values than those who remained clinically stable (p<0.01) [100].

In patients with left ventricular assist devices (LVADs) we found that TDI is useful in the evaluation of myocardial recovery during mechanical unloading [101]. The more recent introduction of 2D-strain imaging in our weaning protocol for patients with mechanical circulatory support has substantially improved our ability to evaluate cardiac recovery during mechanical unloading and the information obtained (global strain and strain rate plus evaluation of mechanical synchrony and synergy) was essential to the decision to wean six patients from their assist devices. To date none of these 6 patients showed heart failure recurrence after assist device explantation. Fig. (**10**) shows the time course of 2D-strain images recorded during reduction of the LVAD (Type Incor) rate in a patient with idiopathic dilated cardiomyopathy who showed relevant myocardial recovery during mechanical unloading.

Although strain rate imaging provides several details on diastolic  ventricular  function, the clinical  value of diastolic strain and strain rate parameters was less investigated. Wall motion velocity measurements with pulsed-wave tissue Doppler revealed high predictive values for acute cardiac allograft rejection of early diastolic wall motion peak velocity (Em) reduction, prolongation of early diastolic time (TEm = from onset of relaxation to the peak of the early diastolic wave Em) and reduction of Em/TEm ratio [77]. Preliminary results of our recently started investigation on the clinical value of cardiac rejection surveillance with 2D strain imaging also revealed a significant reduction in early diastolic strain rate (DSR_E_) and prolongation of the time from onset of relaxation to the peak of DSR_E _during acute cardiac rejection, suggesting the usefulness of diastolic strain parameters as markers for early non-invasive detection of cardiac rejection [40]. The assessment of diastolic function by 2D-strain imaging appeared also useful for the evaluation of patients referred for heart transplantation because parameters like late diastolic strain rate (DSR_A_) and the diastolic E/A strain rate ratio (DSR_E_ /DSR_A_) showed high predictive values for the outcome of patients with idiopathic dilated cardiomyopathy (IDCM) during the first 6 months after listing for HTx [102].

In IDCM patients with similar LVEF and peak oxygen consumption (VO_2_max) at the time of listing for HTx, those who showed rapid clinical worsening had significantly (<0.01) lower DSR_A_ and higher DSR_E_ /DSR_A _ratios than those who remained stable during the first 6 post-listing months [102]. At a cut-off value of <0.3/s the DSR_A _showed high positive and negative predictive values (89% and 90%, respectively) for deterioration of cardiac function during the first 6 post-weaning months which recommend this parameter as a useful tool for listing decisions (elective, urgency or high-urgency) [102]. 

A next step in development of deformation imaging by echocardiography which will be superior to the 2D strain and SR imaging will be the calculation of strain and SR in three dimensions during the same heart cycle. The potential diagnostic benefits of this further technical development remain, however, to be established. 

## CONCLUSIONS

Echocardiographic strain and strain-rate imaging is a promising tool for the evaluation of myocardial function. The spectrum of potential clinical applications is very wide due its ability to differentiate between active and passive movement of myocardial segments, to quantify intra-ventricular dyssynchrony and to evaluate components of myocardial function such as longitudinal myocardial shortening that are not visually assessable. The high sensitivity of both TDI-derived and 2D speckle tracking derived strain and strain rate data for the early detection of myocardial dysfunction recommend this new non-invasive diagnostic method for routine clinical use. Speckle tracking derived 2D-strain measurements have the advantage of angle independency but are sensitive to image quality. TDI-derived strain measurements are less sensitive to image quality but the angle dependency of the method is an important limitation. In addition to early detection of myocardial dysfunction of different etiologies, assessment of myocardial viability, detection of acute allograft rejection after HTx and early detection of patients with TxCAD, strain and strain rate measurements are helpful in the selection of different therapies (CRT, coronary revascularization, SVR and HTx). Strain and strain rate data also provide valuable prognostic information, especially for prediction of future reverse remodelling after left ventricular restoration surgery or after CRT and prediction of short and median-term outcome without transplantation or ventricular assist device implantation of patients referred for HTx.

## Figures and Tables

**Fig. (1):**
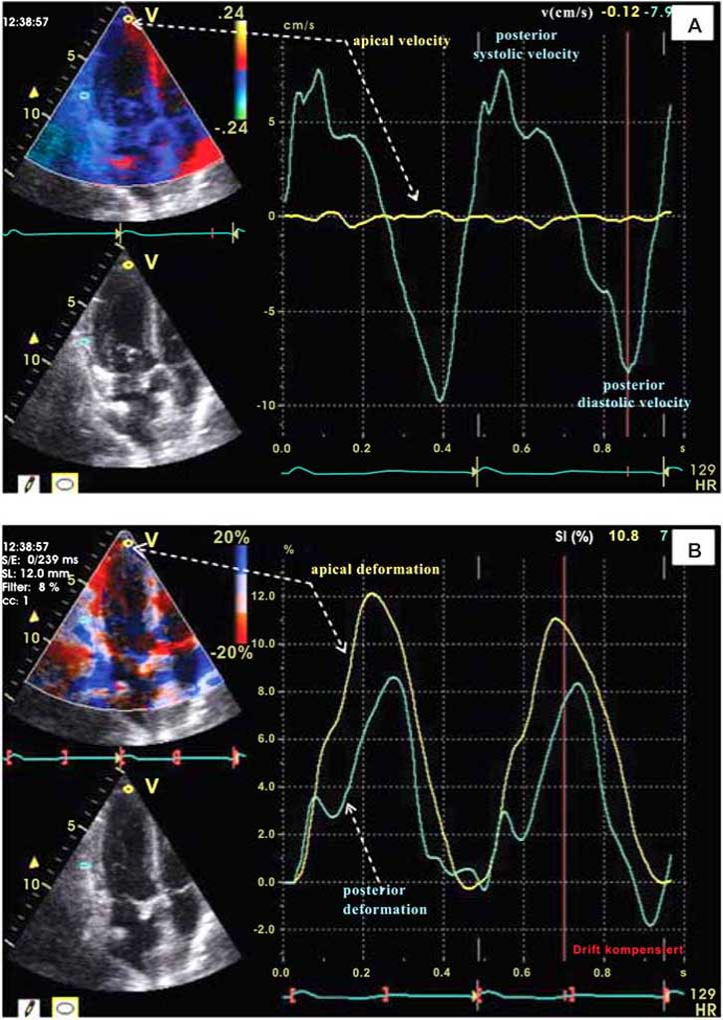
Tissue Doppler derived left ventricular wall motion velocity (panel **A**) and myocardial strain (panel **B**) images from a heart transplanted patient with normal cardiac function and no evidence of coronary artery disease. The velocity and strain curves were obtained in apical long axes views during the same cardiac cycle from the same two myocardial regions (posterior-basal and apical). Because of the velocity gradient which normally exists between the basal and apical LV regions (highest at the base and lowest in apical regions), the assessment of wall motion velocity is not useful for detection of regional differences in contractile function. Thus, as shown by the yellow curves, despite the very low wall motion velocity in the apical region (panel **A**), the longitudinal myocardial shortening in this region can be even higher than in basal regions (panel **B**).

**Fig. (2):**
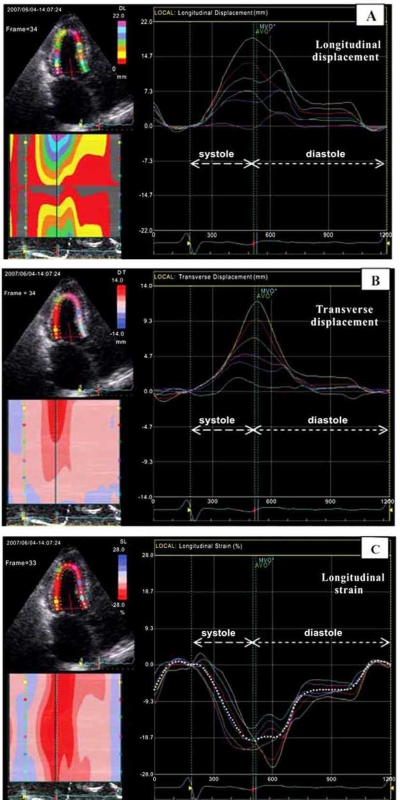
Speckle-tracking 2D-strain imaging (apical long axis view) in a heart transplanted patient with normal LV function and no angiographic evidence of coronary artery disease. The same echocardiographic loop was used for evaluation of myocardial displacement and longitudinal deformation (strain) in the 6 visible LV wall segments. The images in panel **A** and **B** show that myocardial displacement can be misleading by suggesting regional differences in contractile function, although, as shown in panel **C**, myocardial deformation analysis (strain imaging) does not reveal relevant regional differences in myocardial longitudinal shortening.

**Fig. (4):**
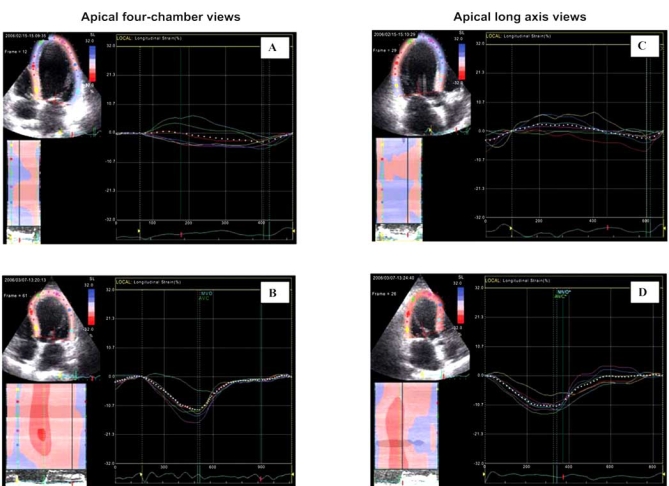
Longitudinal strain before and after cardiac surgery (coronary bypass and mitral valve reconstruction) in a patient with coronary artery disease associated with severe mitral regurgitation. Global strain (white dotted line) increased from less than 3% preoperatively (**A** and **C**) to 11% after surgery (**B** and **D**). There was also a relevant improvement in the synchrony and synergy of regional systolic longitudinal shortening revealed by the more uniform amplitude and time course of the differently colored regional strain curves. [Knosalla C, Dandel M *et al.*, Annual Meeting of the German Society for Thoracic and Vascular Surgery 2007].

**Fig. (5):**
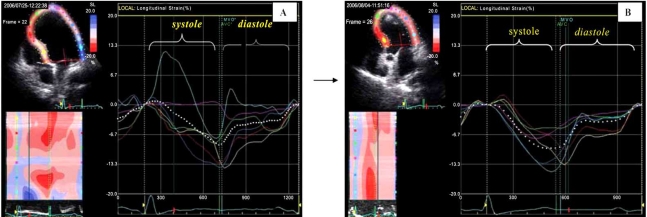
Left ventricular longitudinal strain images obtained from the 4-chamber view of a patient with LV apical aneurysma after myocardial infarction before (panel **A**) and after (panel **B**) surgical LV restoration. Less systolic asynchrony (more uniform contraction), more uniform relaxation and improvement of contractile function in apical and basal lateral regions were the most evident postoperative changes detectable by 2D strain imaging. [Knosalla C, Dandel M, *et al.* Journal of Heart Lung Transplant 2008; 27: S186].

**Fig. (6):**
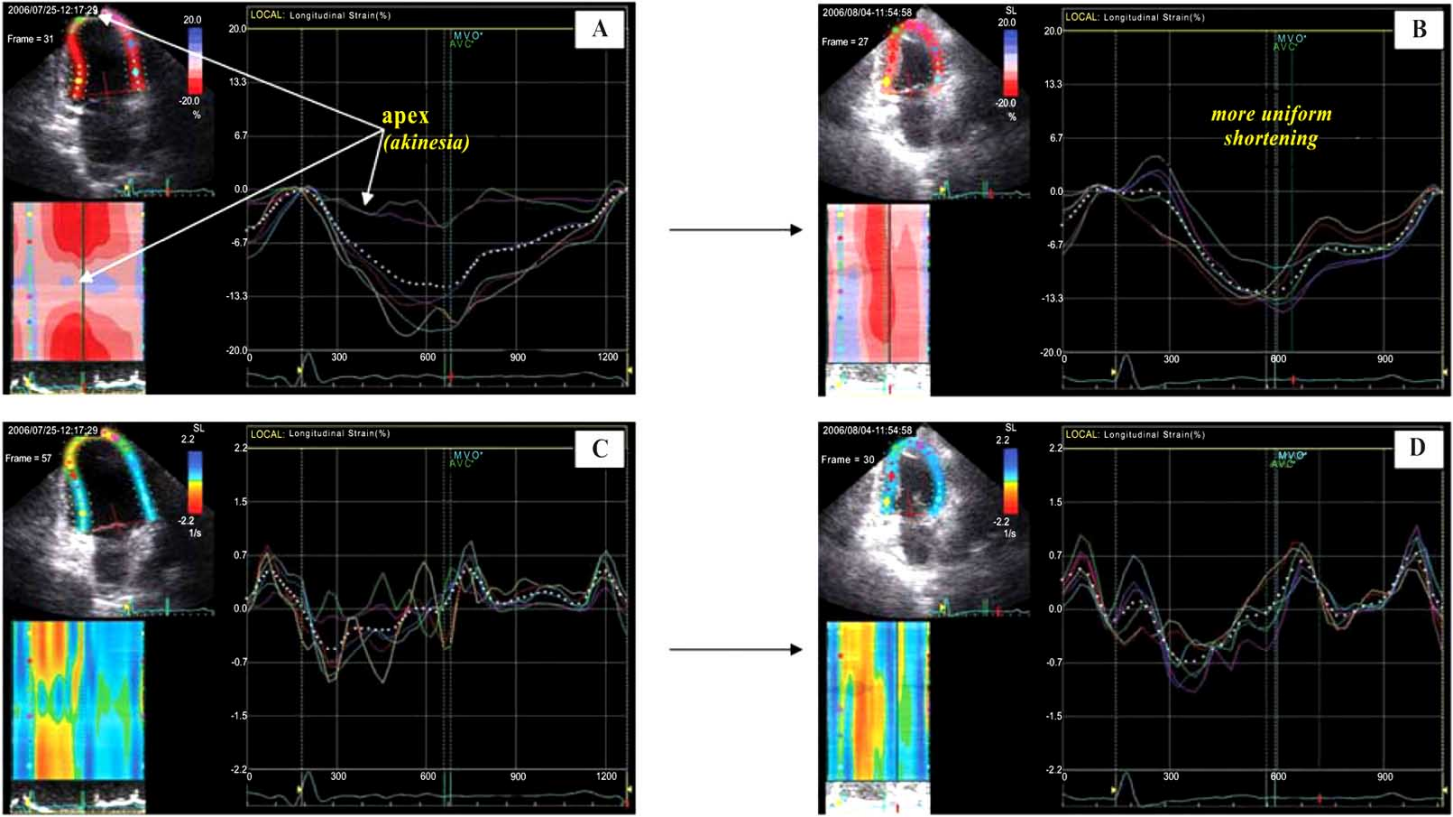
Left ventricular longitudinal strain (shortening) and strain rate (velocity of shortening) images obtained from apical 2-chamber views before (**A** and **C**, respectively) and after surgical ventricular restoration (**B** and **D**, respectively) in a patient with initially severe LV dysfunction after apical myocardial infarction. Panels **B** and **D** show more uniform shortening (amplitude and velocity, respectively) after surgery. [Knosalla C, Dandel M *et al.*, Annual Meeting of the German Society for Thoracic and Vascular Surgery 2007].

**Fig. (7):**
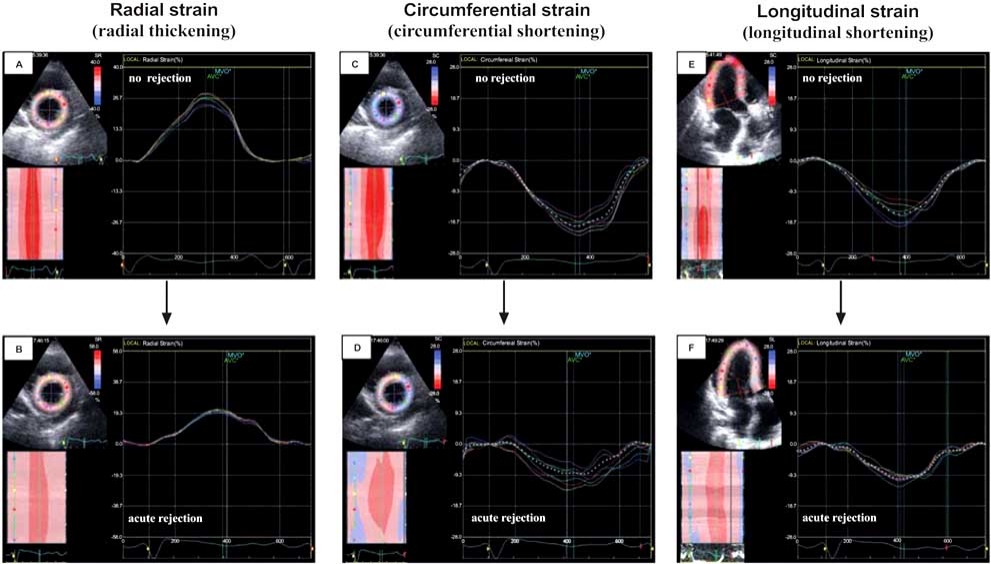
Left ventricular strain changes during symptomatic, biopsy-proven acute rejection (mixed cellular and vascular rejection). Radial (**A** and **B**), circumferential (**C** and **D**) and longitudinal (**E** and **F**) global strain decreased during rejection by 24%, 50% and 38%, respectively, without changes in synchrony and synergy of myocardial contraction. [Dandel *et al.* 2007, oral abstract, AHA Scientific Session].

**Fig. (8):**
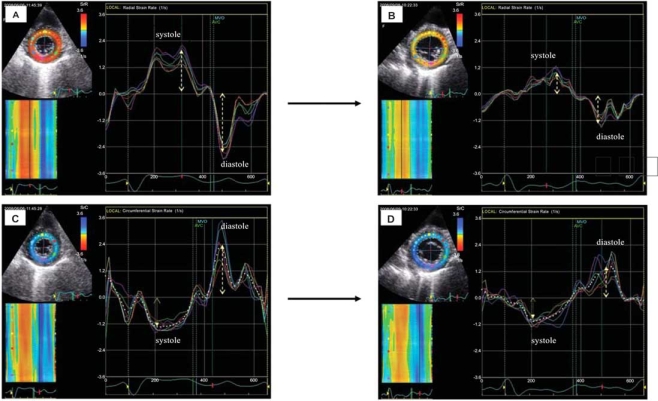
Left ventricular radial (**A** and **B**) and circumferential (**C** and **D**) strain rate changes in an asymptomatic patient with mild acute rejection (ISHLT grade 1). The peak systolic and diastolic strain rates (dotted yellow arrows) were higher in rejection-free state (**A** and **C**) and lower during rejection (**B** and **D**). Strain rate reduction was more evident in diastole than in systole. [Dandel *et al.* 2007, oral abstract, AHA Scientific Sessions].

**Fig. (9):**
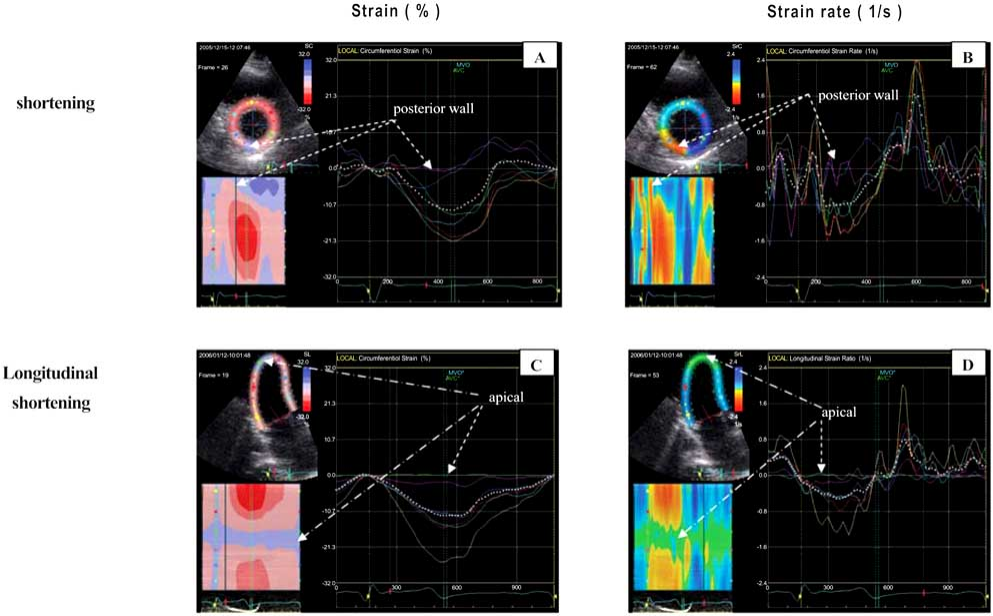
Left ventricular strain and strain rate images in heart transplant recipients with focal stenoses of coronary arteries. **A** and **B**: Circumferential strain and strain rate in a patient with stenosis of the right coronary artery. **C** and **D**: Longitudinal strain and strain rate in a patient with stenosis of the left anterior descending coronary artery. [Dandel *et al.* JHLT 2008; 27(2): S95-96].

**Fig. (10):**
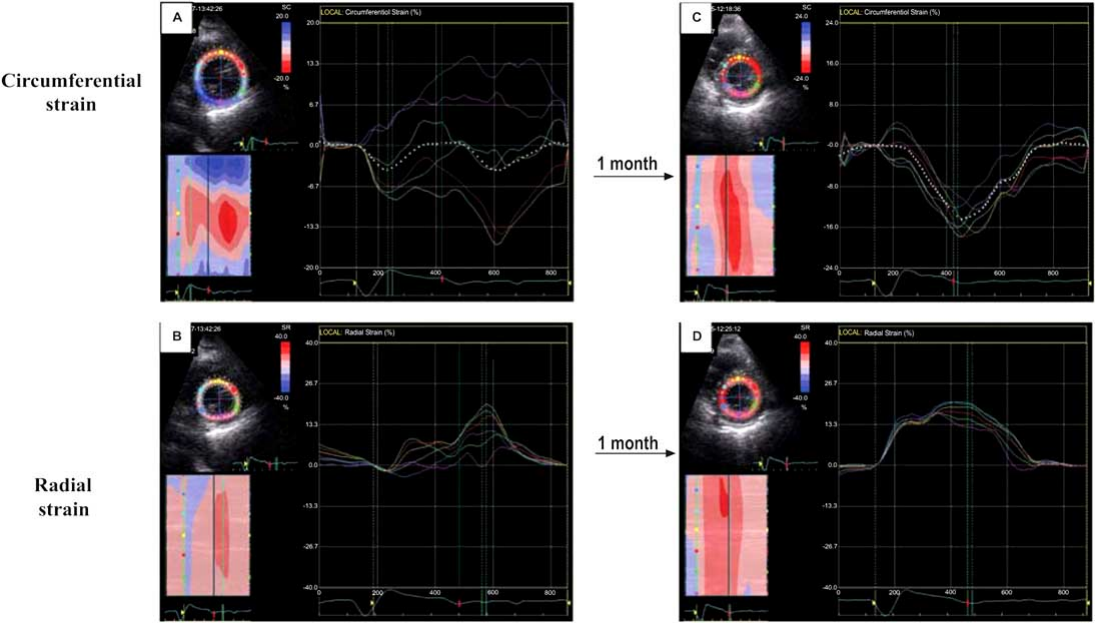
Time course of 2D-strain images recorded during reduction of the LVAD (Type Incor) rate in a patient with idiopathic dilated cardiomyopathy who showed relevant myocardial recovery during mechanical unloading. After LVAD implantation, the left ventricular global strain values were low and regional strain curves showed important dyssynchrony and dyssynergy (**A** and **B**). One month later global strain values were several times higher and regional strain curves indicated uniform circumferential shortening and radial thickening (**C** and **D**, respectively). [Dandel *et al.* 2007, oral abstract, AHA Scientific Sessions].
